# Screening for Autism Spectrum Condition Through Inner City Homeless Services in the Republic of Ireland

**DOI:** 10.1007/s10803-022-05669-x

**Published:** 2022-08-10

**Authors:** A. M. Boilson, A. Churchard, M. Connolly, B. Casey, M. R. Sweeney

**Affiliations:** 1 School of Nursing, Psychotherapy and Community Health, Dublin City University, Glasnevin, Dublin 9, Ireland; 2 Buckinghamshire Older People’s Psychological Services, Oxford Health NHS Foundation Trust, Whiteleaf Centre, Bierton Road, Aylesbury, HP20 1EG UK; 3 Dublin Simon Community, 5 Red Cow Lane Smithfield, Dublin 7, Ireland

**Keywords:** Autism spectrum Condition, Screening, Epidemiology, Homelessness

## Abstract

Homeless service users were screened for autism spectrum disorder through one of Ireland’s leading not for profit service providers. Keyworkers acted as proxy informants; their caseloads were screened using the DSM-5—Autistic Traits in the Homeless Interview (DATHI). Client current and historical health and behaviour data was collated. A representative sample of 106 eligible keyworkers caseloads were screened, identifying 3% “present” and 9% “possibly present” for autistic traits with the DATHI. These findings suggest a high estimate of autism prevalence and support emerging evidence that, people with autism are overrepresented in the homeless population, compared to housed populations. Autism may be a risk factor for entry into homelessness and a challenge to exiting homeless and engaging with relevant services.

## Introduction

Autism is a neurodevelopmental condition characterised by difficulties with social relating, social communication, flexibility and sensory processing deficits (American Psychiatric Association, 2013). The prevalence of autism in Ireland has been estimated as a minimum of 1% of the population (Boilson et al, 2016) but the authors estimate that the true prevalence, allowing for methodological issues is closer to 1.5%. This estimated prevalence rate has been adopted by the Irish Department of Health (2018) for development planning policy and services. The international estimated prevalence of autism spectrum condition (ASC) in adults is 11 per 1000 (Brugha et al., 2016).

There are increasing concerns about the poor long-term outcome for so many individuals with this condition (Howlin & Megiati, 2017). It is estimated that 70% of people with ASC experience at least one comorbid psychiatric disorder (Buck et al., 2014). Iemmi, Knapp, & Ragan (2017) considered the lifelong incidence of mental health difficulties among people with ASC to be as high as 80%. Forty percent of autistic individuals may have two or more psychiatric disorders (DeFilippis, 2018) primarily depression and anxiety disorders. Mental health issues among the homeless population can result from adverse childhood experiences (Haruvi-Lamdan et al., 2018) and the challenges of living with autism (Fitzpatrick et al., 2016). People with ASC are more vulnerable due to family and relationship breakdown, difficulties in communication skills, social naiveté difficulties, recognising and reporting/addressing bullying or adverse experiences. High levels of bullying among adolescent students with ASC was identified in a systematic literature review by Malano et al., (2016) & Forrest et al. (2020). Other studies highlight that bullying, exploitation and victimisation often continues into the adult lives of people with ASC (Lougheed & Farrell, 2013; Paterson et al., 2012). Butwicka et al., (2017) highlight increasing prevalence and under diagnosis of substance use among people with ASC. Lundström et al., (2011) found that participants diagnosed with ASD were six times more likely to be at risk of substance use than those without ASC. However, Ressel et al., (2020) highlight limited evidence for the prevalence of substance use among people with ASC. The reported prevalence rates of co-existing ASC and substance abuse are as high as 19% −30% (Hofvander et al., 2009; Sizoo et al., 2010). Butwicka et al., (2017) identified adverse childhood experiences among people with ASC and ADHD as significant risk factors for developing substance abuse.

Clarke et al. (2016) highlighted the tendency of people with ASC to use alcohol to self-medicate and relieve anxiety. The complex presentations of these comorbidities makes diagnosing co-occurring psychiatric disorders more challenging for mental health service providers (Hossain et al., 2020). Therefore, it is essential to understand the inter-relationships between mental illness and trauma to ensure sensitive diagnosis and support interventions, effective policymaking and capacity building across health systems to improve social and healthcare (Baxter et al., 2015).

Autistic adults are at increased risk of homelessness due to the association between poor socio-economic outcomes and discrimination (Stone, 2019). Assessing rates of autism among homeless adults is challenging because of the existing methodological difficulties in diagnosing adult autism, coupled with the task of accessing and engaging this “hard to reach” population. Lai and Baron-Cohen (2015) refer to this population of undiagnosed adult autistics as a ‘lost generation,’ because of their lack of awareness that autism may account for psychosocial challenges. Churchard et al (2019) estimated the prevalence of autism among a sample of long term homeless people in the UK as 12% in comparison with the prevalence of autism in the general population as 1% −1.6% (Brugha et al., 2016; Rydzewska et al., 2018). Churchard et al’s (2019) study is the most methodologically robust estimates of autism prevalence in any adult homeless population. These prevalence rates warrant further investigation to understand the relationships between autism and homelessness. It is important to emphasize that homelessness is not an outcome of autism, but one of the disabling barriers autistic adults face throughout their lives’ (Stone, 2019). These barriers which can include social isolation, poor communication skills, lack of community understanding and support, employment disadvantage and discrimination are likely to be key reasons why autistic adults may be more at risk of homelessness (Copper et al., 2017).

Previous research has highlighted high rates of autistic people (often undiagnosed) amongst those who are homeless (Evans, 2011; Pritchard, 2010). These findings are understandable given the confusing and overwhelming housing support environments and the desire of many people with autism to withdraw from congregations to manage their own space. The negative sequel of entrenched homelessness for rough sleepers on the spectrum can include deterioration of physical and mental health, addiction, bullying, harassment, sexual/physical abuse, economic exploitation (false befriending with the aim of extortion) and hate crime (Canavan, 2018; Stone, 2018).

Kargas et al., (2019) assert that, paradoxically, living on the streets may offer a place of relative safety from abuse and exploitation. Pritchard’s (2010) found that while there is often support for people with autism and intellectual disability through adult services, those with a diagnosis of high functioning autism were more likely to not be in receipt of support and at a higher risk of homelessness than the general population. Social care staff may not have adequate training, knowledge and skills concerning the characteristics and support needs of people with autism; often interpreting client difficulties as mental illness (Campbell et al., 2015). When distress is exhibited through unusual or challenging behaviour, autistic people can be labelled as uncooperative, oppositional, or anti-social (Lougheed & Farrell, 2013). Consequently, homeless individuals population on the autism spectrum may not receive screening, support and be allocated inappropriate accommodation. The aim of this study was to estimate the prevalence of autism spectrum condition in the homeless population in Dublin, within a specific catchment area. This was undertaken as part of a larger study which will be reported separately which explored the skills, knowledge and training needs of keyworkers nationally in relation to autistic clients and also explored the lived experiences of a small number of people who were autistic and experiencing homelessness.

## Methodology

This study was carried out in partnership with one of Ireland’s leading not for profit homeless service providers, Dublin Simon Community. The majority of homelessness in the Republic of Ireland occurs in Dublin, Ireland’s largest city. The total population of Ireland is estimated at 5.01 million with 1,426,000 based in Dublin (CSO, 2021). 6131 adults in Ireland are homeless and of these 4000 adults are based in Dublin (Dublin Simon, 2021). This sample was drawn from a typical region in Dublin City Centre, catered for by the Dublin Simon Homeless agency.

The study team implemented the methodology developed by Churchard et al (2019) who conducted an autism prevalence study in a London based homeless population. The UK study used a quantitative cross-sectional study design based on researcher administered in-depth structured interviews with key workers who provided proxy information about their client base. Keyworkers in the current study were based in a Dublin City Centre area and provided a range of homeless services; emergency accommodation, supported housing, treatment and recovery services.

### Sample & Recruitment

Key workers with established relationships and good levels of knowledge of their client’s caseload were recruited by the service’s research and advocacy officer and managers of the targeted services. The study team disseminated information related to the studies objectives, methodology, screening criteria, materials i.e., consent forms, plain language statement to the targeted keyworkers through service managers, requesting their participation in the study. The first author performed training sessions (approximately 2 h in duration) with keyworkers who provided consent to participate in the study in advance of the data collection. The objectives of the training sessions were to provide keyworkers with an overview of common autistic traits and explain the screening tools that would be used in the one-to-one structured interviews. Keyworkers had the opportunity to ask questions and request clarification on all aspects of the study methodology, screening protocol, study materials, confidentiality, client anonymity and ethical issues. In advance of the training keyworkers had varying degrees of knowledge depending on their own professional and personal backgrounds. Each keyworker’s entire caseload was included in the study sample, provided they met the study criteria.

### Measures

We used the *DSM-5-based Semi-Structured Interview (DATHI) *developed and validated by (Churchard et al., 2019). Keyworker’s knowledge of their client’s characteristics was served as a proxy for the identification of homeless clients with traits of autism. The screen facilitated the study team to capture knowledge related to each client’s presentation. The screen has separate sections for each of the seven criteria, with general questions followed by specific prompts. Some questions were adapted to the homelessness context, based on the information gained from experts in the development phase. The DATHI scores obtained indicated whether autism symptoms were present for each of the seven DSM-5 criteria. A range of scoring options were used including ‘*possibly present*’, ‘*not present*’, ‘*present* and ‘*insufficient information to classify*’. Scores on individual criteria were combined to make an estimated overall classification for each homeless client. There were four possible summary outcomes: (1) *screened positive–high likelihood of DSM-5 autism* (2) *marginal–medium likelihood of DSM-5 autism* (3) *screened negative–low likelihood of DSM-5 autism* (4) *unclassified–insufficient information*.

Two questionnaires from the UK study were used to gather information relating to: Questionnaire 1—*Demographics and health status and Questionnaire 2—behaviour history and Client characteristics and support needs*. The first  questionnaire captured information relate to the client demographics, nature of homelessness, current accommodation situation, mental and physical health history, presence/absence of neurodevelopmental disorders, drug and alcohol history, risk to self (self-harm, self-neglect, failure to comply with medication) and risk to others/from others (violence and aggression, financial exploitation), history of health and social care service usage prior to age of 18 years, history of service relationships.

The second questionnaire captured background information about the client’s history of homelessness, current and past relationships (inside & outside the service), relationship breakdowns, barriers obtaining stable accommodation, skills & obstacles for achieving independence. The client’s views of their current situation, goals, priorities, strengths were also captured in this questionnaire. Keyworkers experiences in assisting clients to manage their personal challenges and the factors hindering their progress towards achieving independent living were also taking into consideration as part of the screening process.

### Data Collection Process

The systematic data collection process required keyworkers to act as informants for their eligible client caseload. In homeless services in the Republic of Ireland and in the UK each homeless person is assigned a keyworker, a member of staff who coordinates their contact with services and who works directly with their own client caseload. Homeless clients born outside of the United Kingdom and the Republic of Ireland were not eligible to be included in the study. The characterisation of the homeless population born outside of these regions is a distinct phenomenon, in terms of its causes (Fitzpatrick et al., 2012; Phillips, 2006). During the training session Keyworkers were provided with a copy of the study materials (information sheet and consent form) and were given an opportunity to ask any questions arising. To protect the privacy and confidentiality of the keyworker caseload, the DCU study team did not have access to client identifiers (names and dates of birth). A master tracking log was kept by the research officer at the homeless service provider, whereby a unique identifier was assigned to each keyworker client caseload. The core research team and the research officer at the homeless service liaised continuously throughout the data collection process to ensure that clients who moved across services i.e., treatment and recovery to temporary accommodation were only interviewed on one occasion and that a representative proportion of clients were screened across the different services. Interviews were scheduled in advance with the keyworkers. This was an essential quality control measure to facilitate each keyworker to review these clients’ case files in advance of the data collection. The study received ethical approval from the University’s Research Ethics Committee.

### Data Analysis

**Fig. 1:**
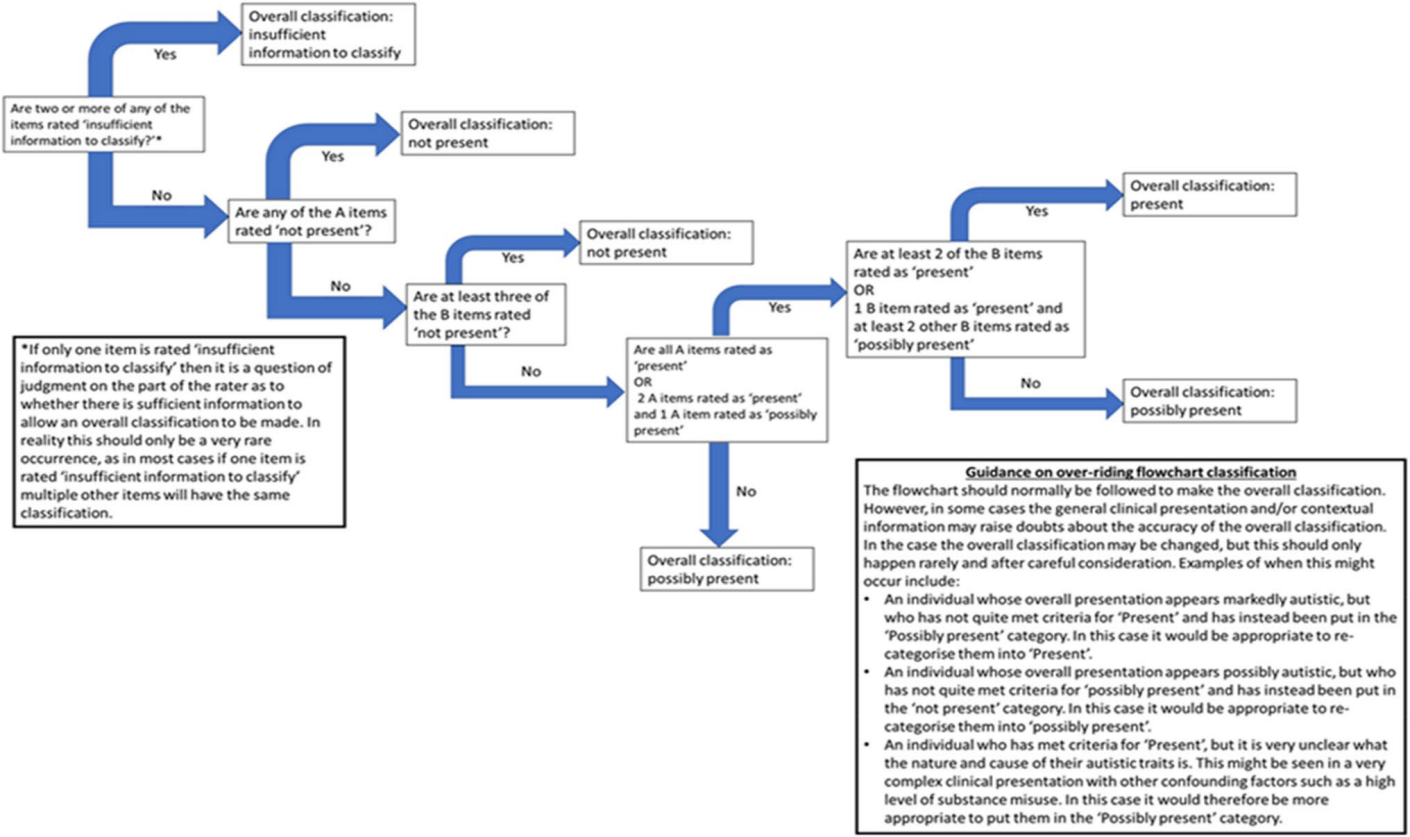
DATHI coding flow chart

The DATHI screening data for each client was scored manually, rating whether autism symptoms were present for each of the seven DSM-V criteria. The four scoring options were as follows: “*present*” “*possibly present*”, ‘*not present*’ or ‘*insufficient information*”. Sub scores were combined to make overall classifications for each client. After the scores were independently calculated by the research team based in Dublin the scores were further validated by a Clinical Psychologist on the team, based in London, to ensure that the sub scores and overall categories were correctly assigned according to the scoring criteria, see Fig. 1—*DATHI Coding Flowchart*. The validated caseload screening, descriptive data, demographics, heath, behaviour history client support needs were evaluated in Excel and IBM SPSS™ v 25.

## Results

### Keyworker Response Rates

**Fig. 2:**
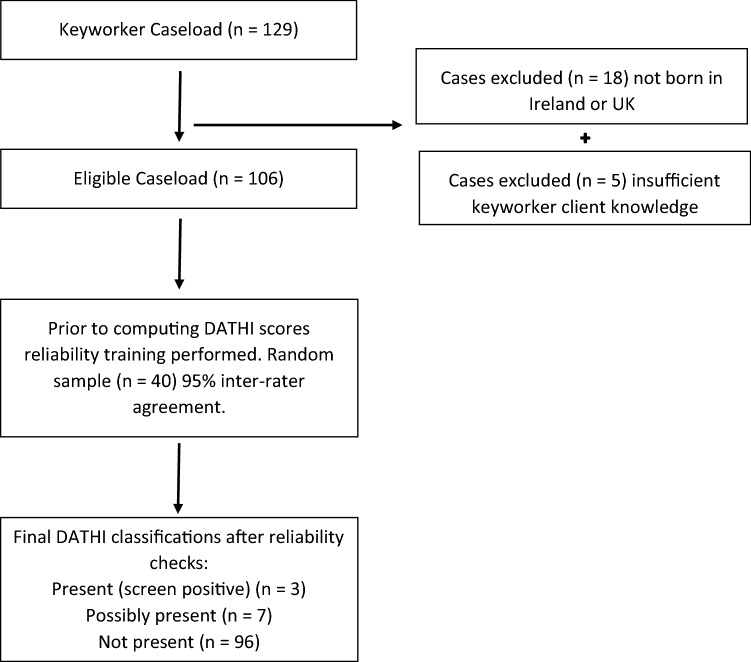
Keyworker caseload screening classification process

We obtained a representative sample across the different areas of service provision offered by the homeless service involved; these included—emergency services, supported housing and treatment and recovery. We invited all 14 key workers who worked with a national homeless service provider based in a metropolitan locality to participate. Eleven of the 14 agreed to participate, giving a response rate of 78%, and providing data for 106 clients, in a structured format. The distribution of these 106 clients across the areas of homeless service provision were as follows: emergency services (n = 42, 40%) supported housing (n = 37, 35%) and treatment and recovery services (n = 27, 25%). Seventy-eight percent (n = 83) of clients were males, 22% were females (n = 23). The screening process is provided in Fig. 2—*Keyworker Caseload Screening Processes.* Keyworkers had worked with their client caseload on average for 15 months, (median 8.5 months). The average key worker’s caseload was 10 clients, ranging from 4–11 clients.

### Client Characteristics

Fifty-five percent of keyworkers (n = 58) had meetings with their clients weekly, 30% had meetings fortnightly (n = 32) and 13% had meetings daily (n = 14). Ninety-six percent of clients were white Irish (n = 102), 3% were white Traveller (n = 3) and 1% were black Irish (n = 1). The age range of clients was 19–77 years, (median age 45 years). The average length of time clients was homeless was 7.6 years, (SD 6.8 years, median 5 years). There were no significant differences observed in length of time homeless and client gender. Seventeen percent of clients had engaged with homeless services prior to eighteen years of age. This cohort of clients had engaged with adoption & foster care (n = 12, 11%), social services (n = 18, 17%), local authority care (n = 3, 3%), young offenders’ institutions (n = 9, 8%) and mental health services (n = 3, 3%). Further details of the client’s backgrounds, childhood experiences and risk factors are captured in a separate publication emerging from this study. This is preparation at this time (Casey B et al., 2022).

### Coding

The DATHI coding process was discussed previously in the methodology section of for this study. Keyworkers considered and scored the following items (See Table 1, A1–B4) about each of their clients in a researcher administered structured interview.

Sample keyworker responses are illustrated in Table 1.

**Table 1:** sample of keyworkers responses scoring clients as present, possibly present on the DSM-V autistic traits on the homeless interview (DATHI)

Criterion	Examples of behaviours considered with autistic traits		
A1—Deficits in social emotional reciprocity	The client will not initiate social contact, only converse very brief contact with others. Looks at the floor in conversation with staff, very little eye contact. Does not discuss feelings, but when in conversation is completely immersed	Does his own thing, will not engage with other people unless approached. Monosyllabic replies/Monopolises conversation	Absence of greetings. Responds only to question, does not engage in back/forth conversation
A2—Deficits in non verbal communicative behaviours used for social interaction	The client has a fixed gaze when conserving with keyworker	Fixed gaze, aggressive when he is placed in the position of having one to one contact. Facial expressions absent, keyworker stated would not know what he is thinking from the expression on his face, given lack of expression	The client does not care about the impact of his behaviour on others, no interest getting on with staff in the current service, hostile, suspicious, trust issues, no interest in engagement with others, has no cliques in or outside of the service, very isolated
A1—Deficits in developing, maintaining & understanding relationships	The client has limited engagement, brief interactions with staff	Client does not engage, according to keyworker is in his own world. Will only engage if spoken to, yes/no responses	The client has no interest in contacting anyone within the service, for example other clients, just wants to be left alone. The keyworker thought this behaviour was functional.
B1—Stereotyped, repetitive motor movements, use of objects or speech	Shakes legs when sitting and in discussion with staff, monotonous, formal tone of voice	The client paces a lot, even in own room of the house constantly walking back and forth… robotic, no feeling to the tone of client’s voice	Pacing back and forth a lot in room, never sitting according to keyworker, intonation of speech unchanging. Goes off on tangents in conversation
B2—Instances of sameness, inflexible adherence to routines or ritualized patterns of verbal or non verbal behaviour	The client has a fixed routine, going to and getting back from clinic, gets upset if this routine is changed	Isolated routine. Just watches Netflix in his service accommodation, only other routine, going out to get drugs. Isolates himself from other clients in the service. If his phone charge went missing/requested by staff to change room, he gets extremely aggressive. Personal space, territorial, does not want to be disturbed	Does not allow staff into his room when he is not there to supervise. Staff wanted him to change his locker as it was very old, hoards many objects, was resistant to changing his locker. Organisation of belongings—paranoid about people taking them, hence does not allow staff into his room unless he can supervise their activities
B3—Highly restricted fixated interests that are abnormal in intensity or focus	The client constantly discusses a grant he has applied for with staff/keyworker; will not talk about anything else	Discusses sexual topics in conversation and acting out sexually inappropriate behaviour, boundary issues—proposing to random women in the street	The client’s room is very dark, keeps curtains closed, does not like bright light. Likes to touch people, even though he knows it is not correct
B4—Hyper or hypo reactivity to sensory inputs or unusual interest in sensory aspects of the environment	Eating habits, the client does not eat full/regular meals, only sweet foods	Eating behaviour. Diet consists of sweet stuffs, does not eat regular meals and gets very distressed if woken up at any time	The client will wear numerous layers of clothes regardless of the weather, possibly relates to long term rough sleeping

### Score Distribution

**Fig. 3:**
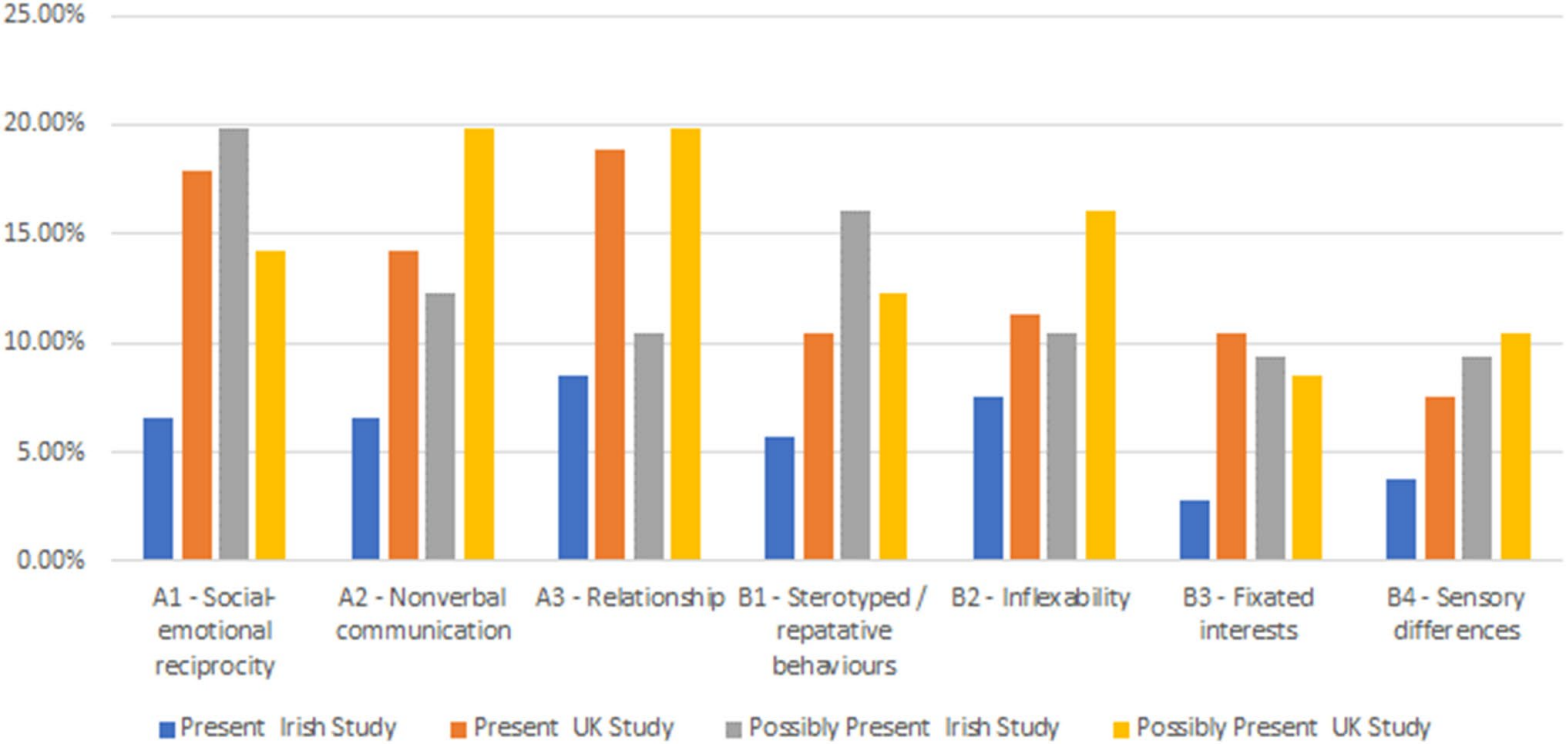
Comparative distribution of DATHI present/possibly present scores for the Irish & UK Studies

The distribution of DATHI’s items (each of which corresponds to a DSM-5 criterion for autism) for the current population of keyworker client’s caseload screened and the UK study are presented in Fig. 3—*Comparative Distribution of DATHI Present/Possibly Present Scores for Irish & UK Studies*. A significantly greater percentage of the UK study clients had elevated DSM V domain scores i.e. (A1)—social-emotional reciprocity (UK/Ireland) (18% /7%), (A2)—nonverbal communication (14%/7%), (A3)—relationship (19%/8%), except for B4—sensory differences (4% / 7%). The same trend was observed for clients who obtained “*possibly present*” scores across the domains A2—nonverbal communication (20%/12%), A3—relationship (20%/10%), B2—inflexibility (16%/10%) and sensory differences (10%/9%), except for A1—social-emotional reciprocity (19%/ 14%), fixated interests (9%/8%) were elevated across the Irish study.

### Screening

Eleven Keyworkers provided information on a total sample of 106 clients. The DATHI sores were then calculated to identify clients with autistic traits including those with *present* and *possibly present* classifications. Three clients (2 males, 1 female) screened *present* for autistic traits, giving a prevalence estimate of 3%. These 3 clients were identified in the keyworkers questionnaire as suspected autism. One additional client was identified by keyworkers in the keyworkers questionnaire as suspected autism (but not classified by the DATHI screening as either *present* or *possibly present). *This client had a keyworker reported diagnosis of schizophrenia. A further 7 clients (6 males, 1 female) screened *possibly present* for autistic traits giving a prevalence estimate of 9% (95% CI: 5% −16% (3 + 7/106) Further details on duration of homelessness are shown in Table 2—*Demographic characteristics and length of homelessness by classification on DSM—5 Autistic Traits in the Homeless Interview (DATHI).*

**Table 2:** Demographic characteristics & length of homelessness by classification on DSM—V autistic traits on the homelessness interview (DATHI)

DATHI classification	Gender		Mean age (SD) in years	Mean length of homelessness in years (SD)
	Male	Female		
Screened positive/present	2	1	45.67 (7.37)	7.67 (5.13)
Screened positive/possibly present	6	1	44.86 (13.11)	11.93 (10.79)
Screened not present	75	21	46.47 (13.17)	7.29 (6.50)

### Physical Health

Keyworker interviews collated data relating to client’s history of mental and physical health difficulties. Fifty-six percent (n = 59) of clients had diagnosed health conditions. The cohort of clients screened had a diagnosis of asthma/COPD (n = 13, 22%), liver/kidney disease (n = 10, 17%), epilepsy (n = 6, 10%), cancer (n = 5, 8%), diabetes (n = 5, 8%), dementia (n = 4, 7%) acquired brain injury (n = 4, 7%), heart disease / stroke (n = 4, 7%) and other conditions (n = 5, 8%).

### Mental Health

Keyworkers reported that 41% of the overall sample (n = 43) of clients had a history of drug/alcohol abuse and (n = 56) that 53% had experienced traumatic events. Fifty eight percent (n = 61) of keyworker clients screened had an underlying diagnosis of mental health problems, which included 67% with depression (n = 41), 34% with an anxiety disorder (n = 21), 23% with psychosis (n = 14), 8% with PTSD (n = 5), 5% with bipolar disorder (n = 3) and 2% with a personality disorder (n = 1). Keyworker’s clients experienced suicidal thoughts (n = 31, 51%), suicidal attempts (n = 13, 21%) self-harm (n = 15, 25%) and neglect (n = 21, 34%). Twenty percent (n = 12) of this cohort of clients experienced difficulties adhering to prescribed medication and non-adherence to medical appointments (n = 16, 26%). Further, these clients were at risk from others with respect to violence & aggression (n = 22, 29%) and psychological abuse & bullying (n = 18, 29%). Keyworkers highlighted problematic behaviour of these clients over the past three months relating to argumentative and abusive behaviour (n = 20, 33%), anti-social behaviour (n = 13, 21%), seeking attention, pestering staff (n = 6, 10%) and physical aggression (n = 5, 8%).

### Neuro-Developmental Disorders

Thirteen percent (n = 13) of the keyworker’s caseload had a diagnosis of a neuro-developmental condition: acquired brain injury (n = 4, 31%) dyslexia/dyscalculia (n = 3, 23%) and intellectual disability (n = 3, 23%). The keyworkers did not identify any clients with a diagnosis of ASC. However, they suspected (n = 4, 4%) had an underlying ASC. Through the DATHI screening process) 3 clients were identified with “*present”* and 7 clients with “*possibly present*” autistic characteristics. These three clients had co-comorbid diagnosis of depression and anxiety disorders. The remaining 7 clients who were identified with “*probably present*” autistic characteristics had keyworker reported diagnosis of psychosis, depression, anxiety and attention deficit disorder (see Table 3). It is likely these clients had undiagnosed ASC of various degrees of severity. The one client suspected by their keyworker as having an underlying ASC was identified with the DATHI as “not present”, this client had a reported diagnosis of schizophrenia. The clients who obtained DATHI “*present”* and “*possibly present*” scores were at risk of self-neglect (n = 6, 60%) poor adherence to prescribed medication (n = 6, 60%) and non-attendance of medical appointments (n = 4, 40%). Keyworkers highlighted that 40% of clients (n = 4) had a history of suicide attempts and 30% a history of self-harm (n = 3). Thirty percent of clients (n = 3) were at risk of violence and aggression from others (both inside and outside their accommodation setting). Twenty percent of clients were at risk of financial exploitation (n = 2) and psychological abuse & bullying.

**Table 3:** Co-occurring conditions DATHI classifications of “possibly present” autistic characteristics

Gender	DATHI classifications	Mental health history
Male	Possibly present	Suspected: Psychosis, Autism Spectrum Disorder
Male	Possibly present	Diagnosed: Dyslexia,; Suspected: ADHD
Female	Possibly present	Diagnosed: Depression, Anxiety Disorder. Suspected: Psychosis
Male	Possibly present	Diagnosed: Depression: Suspected OCD, Eating Disorder, ADHD / Autism Spectrum Disorder
Male	Possibly present	Diagnosis ADHD, Suspected: Depression, Anxiety Disorder
Male	Possibly present	Diagnosed: Psychosis
Male	Possibly present	Diagnosed: Anxiety Disorder, Suspected ASD, ADHD

The behaviour patterns of these clients over the past three months were unremarkable with respect to displays of physical aggression, destruction of property and self-injurious behaviour. However, these clients’ key workers reported argumentative, verbal abuse (n = 5, 50%) and anti-social behaviour (n = 4, 40%) was characteristic of these clients with autistic traits.

### Service Utilization

We explored client’s current links with other agencies. The majority were registered with a general practitioner (n = 100, 95%) engaged with drug and alcohol services (n = 43, 41%), community health mental teams (n = 23, 22%) and were assigned to a social worker (n = 18, 17%). Twenty-six percent (n = 28) of keyworker clients were engaged with community employment (CE) schemes, homeless action teams, probation officers and counsellors, psychiatrists.

## Discussion

It is well documented that autism prevalence estimation is a challenging exercise even in a “stable” population (Korgan et al., 2018). Until recently, information on the epidemiology of autism was based on childhood studies (Fombonne et al., 2005; Newschaffer et al., 2007). Autism prevalence estimation among homeless populations is an even more complicated endeavour. We sought to investigate the prevalence of autism in the homeless community in an Irish context using the protocol developed in a UK study (Churchard et al., 2019) using keyworkers working in homeless service provision as key informants/proxy providers of screening information. We screened a representative sample of the homeless population through a large provider of homeless services in Dublin. Keyworker’s clients were identified from supported housing, emergency accommodation and treatment and recovery services. Through a systematic screening process, we estimated a prevalence rate of 3% among the homeless population. This is considerably higher than our estimate in the general population (Boilson et al., 2016) which was ~ 1%. The prevalence rates may be higher in the homeless population but there were considerable methodological differences across the two studies. The current study was conducted in adults using a screening instrument which was previously used in one study in London in a relatively transient population and screening was completed by proxy by keyworkers working in the sector. The earlier study by Boilson et al., (2016) was a cross-sectional study of school based children in Ireland where parents completed the Social Communication Questionnaire (SCQ) lifetime form on behalf of their children and the results were validated against clinical records.

Our finding are lower than the prevalence estimate reported in the UK study, which returned a prevalence estimate of 12%. Combining prevalence estimates for *present* and *possibly present* cases in our study gives a total estimate of 9%, in the UK study the combined estimate is closer to 20%. These findings indicate the estimated rate of autism prevalence in the homeless population in the UK is more than double the rate from the Irish study region. However, the findings from both the Irish and UK studies indicate the prevalence of ASC among the homeless population is considerably higher than in the general population and these findings have important implications for provision of specific autism services for this vulnerable population. It is not clear why the prevalence rates in the UK appear to be higher, but it is unlikely to be due to methodological factors. The Irish study team rigorously followed the protocol used in the UK study. The differential in the estimate, between the two studies are likely to be attributed to the composition of the populations screened. The UK study population were homeless for longer with the average duration of homelessness being 12 years, versus it was an average of 7 years in the Irish study. In the UK study 43% of the sample were considered homeless (19% homeless hostels, 9% living in independent housing, 9% semi-independent accommodation and 8% from the prison population) whereas in the Irish homeless population we screened a relatively more stable population, which did not comprise street homeless clients.

The homeless services supporting the Irish based cohort comprised emergency services (40%), supported housing (35%), and treatment and recovery services (25%). These findings indicate that the UK population were a more vulnerable population, with possibly greater mental health challenges and neuro-developmental difficulties. The overall higher *present* scores for the UK population is evidence that the UK population sampled were lower functioning with respect to social and emotional reciprocity, non-verbal communication and *possibly present * traits such as—non-verbal communication, relationships, inflexibility and sensory difficulties. It is important to acknowledge also the structure and topology of homeless services between the two populations sampled. In Ireland emergency’ accommodation is referred to as ‘temporary’ accommodation whereas in large towns and cities in the UK there is a move away from the provision of emergency shelter towards temporary supported housing models. In Ireland and UK these homeless services are moving to mainstream practice (European Observatory of Homelessness, 2018).

There is a limited evidence base relating to the specific challenges of  homeless people with ASCs around navigating complicated healthcare systems (Davis-Berman, 2016), managing unstable housing situations, balancing competing priorities (Rae & Rees, 2015) and in evaluating previous negative experiences with healthcare services and professionals (Shulman et al., 2018). Homeless clients require assistance securing internships, employment and life skills to achieve personal development. Staff employed in homeless services who recognize cognitive impairment in clients may mistakenly attribute problematic behaviours as wilful refusal to adhere to community household rules or to follow through with treatment recommendations (e.g., medical appointments). Standard housing service’s intakes often do not provide a complete assessment of individuals’ cognitive, mental health, physical wellness, and social support networks (Souza et al., 2020).

Fifty eight percent of the homeless population screened had underlying diagnosed mental health conditions i.e., psychosis, personality disorder, depression and anxiety disorder, and a history of suicidal thoughts, attempts and self-harm. Cognitive impairment is highly prevalent among homeless adults (Backer & Howard, 2007; Burra et al., 2009) along with the co-occurrence of anxiety and depression (Russell et al., 2016). Clients screened in the Irish study experienced violence and aggression, psychological abuse, and bullying inside and outside the service. These findings were specifically evident those for clients who obtained *present* and *possibly present* DATHI classifications, who also had poor adherence to prescribed medications and non-attendance of keyworker and medical appointments. These factors need to be taking into consideration with respect to the provision of homeless services staff training, and in accessing relevant medical and social services. While focusing on client individual-level factors is illuminating, without understanding and balancing health system factors, there is a risk that responsibility for accessing services is inappropriately placed with the individual (Harris et al., 2013; Aidala et al., 2016).

Autistic people who are homeless are unlikely to engage with homeless services due to a range of social and personal factors. Indeed, they may not even be aware that autism exists as a condition or be aware of the characteristics. Interestingly, neuro-developmental disorders, such as autism do not feature highly on websites of homeless providers services. There appears to be a gap in thinking, planning and provision. The lack of specific supports is an issue that needs to be addressed by autism advocacy groups. Healthcare and diagnostic services should be accessible for populations experiencing homelessness. Provision of easy to navigate services with clear pathways are essential. These should be coordinated and designed with patients’ needs in mind to meet this population’s complex needs. These services require stable funding, sustainable staffing, coordination of services and facilitate practitioners and staff to use their expertise in the provision of flexible care (Siersbaek et al., 2021). This research highlighted the need for those who work in homeless service provision to be alert to the potential presence of autistic clients with different needs which may need to be considered at organisational and local levels (infrastructure, management and personal approaches) as well as a potential skills/knowledge deficit of keyworkers and the need for autism specific training. Practitioners in homeless services and across interdisciplinary teams require training to support diagnosis, screening and sensitive support taking particular account of the presence of trauma, comorbidities and addiction.

## Conclusion

In terms of practice, training of homeless services keyworkers is essential to recognise the specific traits associated with ASC to provide clients with optimal support, even in the absence of a formal diagnosis. Within the wider policy context, there is a requirement for accessible and integrated housing supports, social-care, social work, medical and diagnostic services for population experiencing homelessness. These services should be easy to navigate with clear pathways, coordinated and designed for this population with complex needs to bridge the gap between research, policy and service provision. The DATHI tool is a research screening tool. We do not recommend its use as an assessment tool. Further research comparing the tool with gold standard assessment methods such as multi-disciplinary team assessment would be required in different homeless populations.

## Limitations

A limitation of this study was the fact that the study screening tool has not been compared directly with a gold standard assessment methodology such as multi-disciplinary clinical assessment as this would not be possible in this hard to reach population. It was difficult to reach a larger sample due to the COVID-19 pandemic and associated public health restrictions in the service at the time of data collection.
